# Effect of vitamin D supplementation versus placebo on recovery delay among COVID-19 Tunisian patients: a randomized-controlled clinical trial

**DOI:** 10.1186/s13063-023-07114-5

**Published:** 2023-02-20

**Authors:** Hela Abroug, Amani Maatouk, Cyrine Bennasrallah, Wafa Dhouib, Manel Ben Fredj, Imen Zemni, Meriem Kacem, Salma Mhalla, Sarra Nouira, Manel Ben Belgacem, Aymen Nasri, Rim Klii, Chawki Loussaief, Nissaf Ben Alya, Ines Bouanene, Asma Belguith Sriha

**Affiliations:** 1 Department of Epidemiology and Preventive Medicine, University Hospital of Monastir, Monastir, Tunisia; 2 Department of Community Medicine, Faculty of Medicine, University of Monastir, Monastir, Tunisia; 3 Research LaboratoryTechnology and Medical Imaging - LTIM - LR12ES06, University of Monastir, Monastir, Tunisia; 4 Laboratory of Microbiology, University of Monastir, Monastir, Tunisia; 5 Department of Family Medicine, University of Monastir, Monastir, Tunisia; 6 Department of Internal Medicine, University of Monastir, Monastir, Tunisia; 7 Department of Infectious Diseases, University of Monastir, Monastir, Tunisia; 8 Ministry of Health, Tunis, Tunisia

**Keywords:** Vitamins, SARS-CoV-2, Adult, Infection, Convalescence, Tunisia

## Abstract

**Introduction:**

The present study aimed to determine the impact of vitamin D supplementation (VDs) on recovery delay among COVID-19 patients.

**Methods:**

We performed a randomized controlled clinical trial at the national COVID-19 containment center in Monastir (Tunisia), from May to August 2020. Simple randomization was done in a 1:1 allocation ratio. We included patients aged more than 18 years who had confirmed reverse transcription-polymerase chain reaction (RT-PCR) and who remained positive on the 14th day. The intervention group received VDs (200,000 IU/1 ml of cholecalciferol); the control group received a placebo treatment (physiological saline (1 ml)). We measured the recovery delay and the cycle threshold (Ct) values in RT-PCR for the severe acute respiratory syndrome coronavirus 2 (SARS-CoV-2). The log-rank test and hazard ratios (HR) were calculated.

**Results:**

A total of 117 patients were enrolled. The mean age was 42.7 years (SD 14). Males represented 55.6%. The median duration of viral RNA conversion was 37 days (95% confidence interval (CI): 29–45.50) in the intervention group and 28 days (95% CI: 23–39) in the placebo group (*p*=0.010). HR was 1.58 (95% CI: 1.09–2.29, *p*=0.015). Ct values revealed a stable trend over time in both groups.

**Conclusion:**

VDs was not associated with a shortened recovery delay when given to patients for whom the RT-PCR remained positive on the 14th day.

**Trial registration:**

This study was approved by the Human Subjects Protection Tunisia center (TN2020-NAT-INS-40) on April 28, 2020, and by ClinicalTrial.gov on May 12, 2021 with approval number ClinicalTrials.gov ID: NCT04883203.

## Introduction

Coronavirus disease 2019 (COVID-19) represents an emerging respiratory infectious disease caused by the severe acute respiratory syndrome coronavirus 2 (SARS-CoV-2) [1, 2]. It was first identified in December 2019 in Wuhan, China [3]. Then, it has rapidly spread all over the world [1, 4]. The COVID-19 pandemic has generated many unproven and exaggerated claims concerning possible treatments. One highly controversial issue has been the role of vitamin D in the prevention and management of COVID-19 [5]. The role of vitamin D in modulating the immune response against viral and bacterial infections particularly in the respiratory tract was well demonstrated by previous studies [4, 6]. The effect of vitamin D on COVID-19 patients remains a controversial subject [7]. Indeed, some studies [8–10] highlighted the significant beneficial effect of vitamin D in reducing mortality and requiring intensive care unit treatment of hospitalized COVID-19 patients. On the other hand, it's demonstrated that vitamin D was not shown as a protective factor against adverse clinical outcomes among COVID-19 patients [11]. The duration of infectivity of COVID-19 has a great importance in the public health strategy and in the practice of infection control in the healthcare facilities [12]. The recovery period is usually around 10 days among patients with mild-to-moderate symptoms and around 15 days among critically ill or immunocompromised patients [12]. A prolonged duration of viral shedding was associated with a high infectivity of the virus and adverse outcomes [13]. The most of earlier studies have explored the impact of the VDs in hospitalized COVID-19 patients and studied in particular the clinical outcomes such as the fatal issue or the need for intensive care. To the finest of the authors’ knowledge, no study has explored the impact of VDs on recovery delay among COVID-19 patients with mild-to-moderate symptoms. The null hypothesis was: Is the recovery delay among COVID-19 patients similar between the VDs group and the placebo group? The aim of the present study was to determine the effect of VDs on recovery delay among COVID-19 patients with a positive control reverse transcriptase-polymerase chain reaction (RT-PCR) on the 14th day following the confirmation of the diagnosis.

## Methods

The present manuscript was conducted according to the guidelines of the Consolidated Standards of Reporting Trials (CONSORTChecklist2010) [14].

### Trial design

We performed a randomized controlled, parallel-group, blinded, clinical trial.

### Participants

We included patients older than 18 years who had confirmed COVID-19, with a positive RT-PCR, and for whom the RT-PCR remained positive on the 14th day. Participants were consecutively recruited according to laboratory results (COVID-19 positive on the 14th day). We did not include pregnant women and patients who refused to participate.

### Settings and location

Intervention and data collection were performed in the national center for COVID-19 confinement. From May to August 2020, this center included all Tunisian patients having COVID-19.

**Fig. 1:**
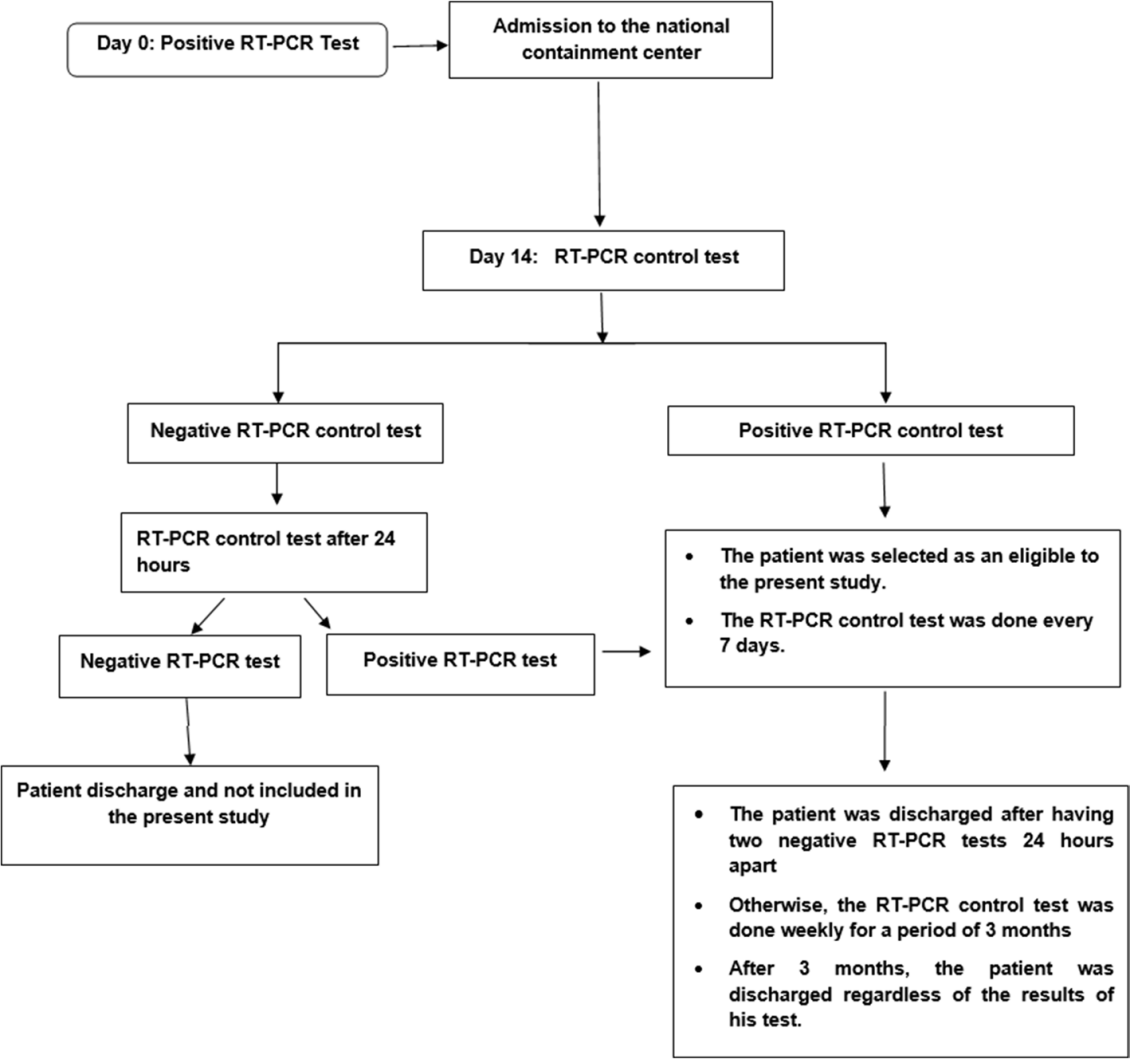
Study protocol flow diagram. Patients with a positive RT-PCR test at 14 days in the containment center were eligible to be included in the present study. The RT-PCR control test was weekly if it remained positive. The maximum period of staying in the center was 3 months. The phone follow-up was done at 1 year and asking about persistent COVID-19 symptoms and a possible second COVID-19 infection after the first episode. RT-PCR, reverse transcription-polymerase chain reaction

At the beginning of the COVID-19 epidemic in Tunisia and to limit the spread of severe acute respiratory syndrome coronavirus 2 (SARS-CoV-2) within the population, the health authorities implemented various interventions such as the creation of a national containment center in Monastir on April 4, 2020. This center was a hotel and allocated 150 beds for patients. Each patient detected COVID-19 positive anywhere in Tunisia, he was transferred by a non-medical ambulance and admitted at this center. The national center was managed by a professor of infectious disease, the preventive medicine department, the bacteriology laboratory, and the psychiatry department. Medical residents and nurses have been assigned to ensure the daily follow-up of the patients. A whole block of this hotel was dedicated to the accommodation of medical and paramedical staff. Each patient was confined to his room and it is forbidden to go out. The meals were given for each in his room. The study protocol is described in Fig. 1.

### Intervention

The intervention started on May and finished on August 2020. Intervention was made by medical residents. Participants were randomized into two groups (Group A and Group B). The intervention group (Group A) received VDs (200,000 IU/1 ml of cholecalciferol (1 ml) oral form), while the control group (Group B) received a placebo treatment (physiological saline (1 ml) oral form). After agreement, medical residents administered the treatment according to group allocation of patients. The containers of the drinkable ampoules containing the vitamin D or the placebo treatment were similar. The treatment was distributed in the same day of the RT-PCR results.

### Outcomes

Our primary outcome measure was the recovery delay defined as the period between the day of the 14th RT-PCR-positive result and the day of the second successive negative RT-PCR test result. Secondary outcomes included the monitoring for the SARS-CoV-2 RT-PCR cycle threshold (Ct) values from the date of randomization until the second successive negative RT-PCR test result. Ct value is inversely correlated to the level of viral ribonucleic acid (RNA) in RT-PCR tests for SARS-CoV-2 [15]. RT-PCR was considered negative if the Ct value was higher than 37, slightly positive if the Ct value was between 33 and 37, and positive if the Ct value was less than 33 [16]. Potential clinical uses of Ct values for SARS-CoV-2 include the assessment of the progression of infection, prediction of disease severity, and determination of contamination [15]. A phone follow-up at 1 year was done.

### Sample size

Sample size was calculated using Package “Medcalc” (trial version). It was estimated in this survival analysis as 118 patients (59 patients in each group). We considered 80% power, alpha = 0.05, and recovery rate at 21 days of intervention: in group 1, 84%; in group 2, 60% [17]. We accounted for a dropout rate of 10%.

### Randomization

Microsoft Excel was used to generate the random allocation sequence [18]. Simple randomization was done in a 1:1 allocation ratio. The principal investigator generated the random allocation sequence, and medical residents enrolled and assigned participants to interventions.

### Blinding

Patients, medical residents, laboratory technicians, and the intervention supervisors were blinded. The groups were labeled as A and B.

### Statistical methods

Data analysis was performed using SPSS Statistics software (SPSS) version 21.0. Statistical analysis was performed using intention-to-treat (ITT) analysis. The Kolmogorov–Smirnov test was used to verify the normal distribution of continuous variables. The mean ± standard deviation (SD) was used to describe the normally distributed data. The median and Inter Quartile Range (IQR) was used to describe the skewed distribution data.

Frequency rates and percentages were used to present categorical variables. The comparison between frequencies was performed using the chi-square test (*χ*^2^ test) or Fisher exact test. Student’s *t* test was used to compare means for two independent samples. The *U*-Mann-Whitney test was used to compare the median duration of viral RNA conversion in the VDs and placebo groups, respectively. The negative conversion of viral RNA indicating recovery during the study period, as time-to-event data, was the outcome measure. Survival analysis using the Kaplan-Meier method was performed to compare the recovery delay between the VDs group and the placebo group. The log-rank test and the Kaplan-Meier curve were used to illustrate this comparison. The Cox proportional hazards model was performed to compare the relative effect of the VDs and the placebo on the recovery delay. Results were reported as hazard ratios (HR) with 95% confidence interval (95%CI). One minus the survival function curve was used to show this effect. A HR value >1 would indicate a prolonged recovery delay, whereas an HR < 1 would mean a short recovery delay. Scatter plots and linear regression were used to evaluate the Ct value trends over time in both intervention groups. The slope of the regression line (b) with the standard error (SE) and Pearson’s correlation coefficient (r) was used to describe the Ct value distribution over time. A *p* value ≤ 0.05 was considered as statistically significant.

### Registration

This study was approved by the Human Subjects Protection Tunisia center (TN2020-NAT-INS-40) and by ClinicalTrial.gov with the approval number ClinicalTrials.gov ID: NCT04883203. The research ethical considerations were respected including free, informed, written, clear and loyal consent, confidentiality, protection, and assistance.

## Results

### Characteristics of participants

**Fig. 2:**
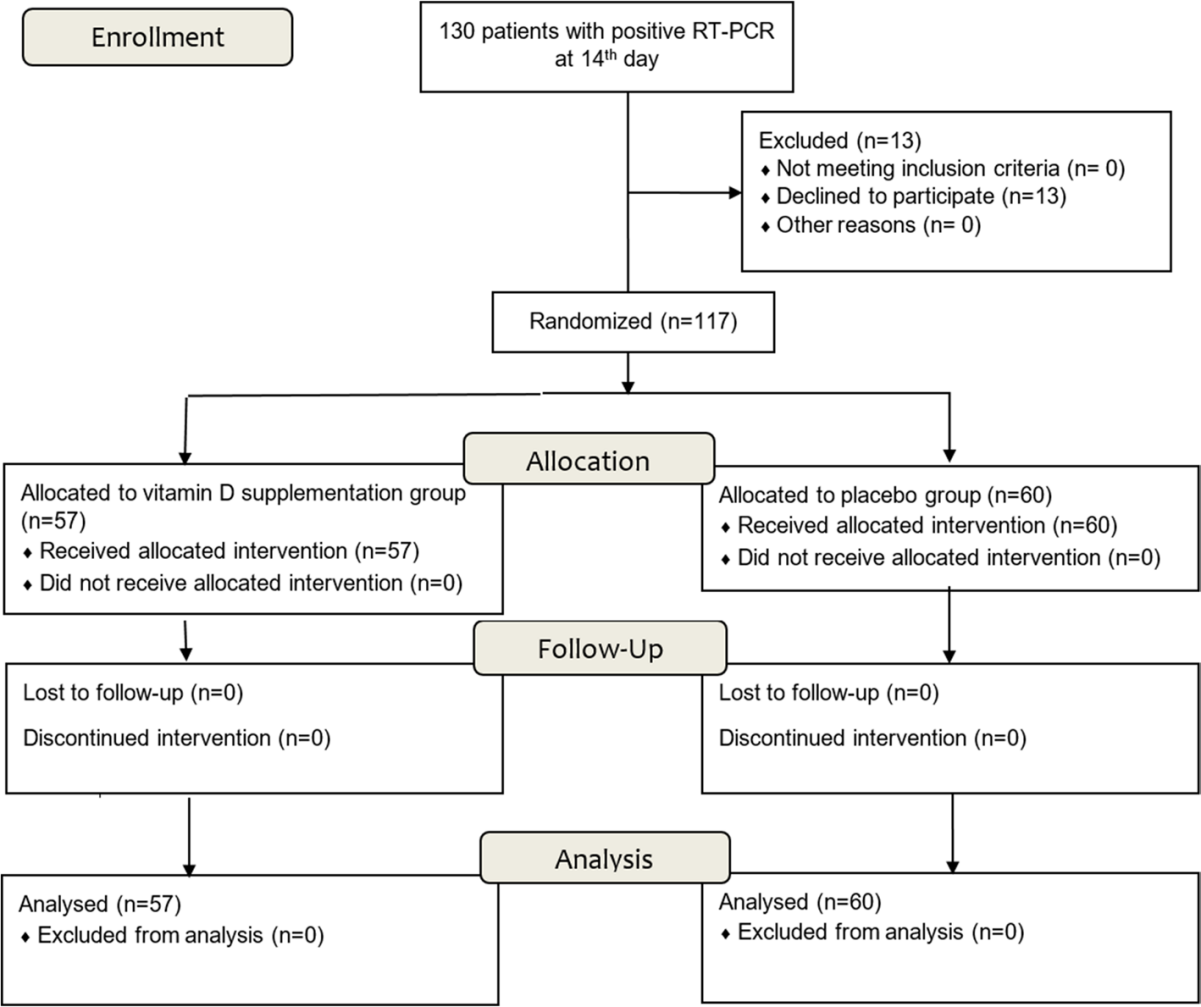
Patients flow diagram. One hundred thirty patients were eligible. A total of 117 patients were randomized into 2 groups: 57 patients (48.7%) in the vitamin D supplementation group and 60 (51.3%) in the control group. RT-PCR, reverse transcription-polymerase chain reaction

A total of 117 participants were enrolled with 57 patients (48.7%) in the VDs group and 60 (51.3%) in the control group (Fig. 2).

The mean age was 42.7 years (SD 14). Males represented 55.6%. The majority of participants (72.64%) had no medical history. The most frequent pre-existing condition was diabetes mellitus (11.96%) followed by asthma and arterial hypertension (6.83% for each one). No significant differences were found between the two groups in all characteristics except for general signs (*p*=0.047). Regarding symptoms, most of the participants were asymptomatic (65.8%), The most common symptoms were otorhinolaryngological symptoms (12.8%) such as anosmia and ageusia and general signs (12.8%) such as asthenia and headache. Means of Ct values before intervention were equivalent for all subgroups (Table 1).

**Table 1:** Participants’ characteristics and Ct values at baseline

	**All participants**	**Intervention group** **(Group A) (*n*=57)**	**Control group (Group B) (*n*=60)**	***p*-value**
***Participants’* characteristics**				
**Age** (years): mean (SD)	42 (14)	43 (15)	41 (14)	0.58
**Sex** (*n* (%))				
Male	65 (55.6)	33 (50.8)	32 (49.2)	
Female	52 (44.4)	24 (46.2)	28 (53.8)	0.62
**Medical history** (*n* (%))				
None	85 (72.6)	36 (42.3)	49 (57.6)	0.06
Asthma	8 (6.8)	3 (37.5)	5 (62.5)	0.26
Diabetes millitus	14 (11.9)	7 (50.0)	7 (50.0)	0.81
Arterial hypertension	8 (6.8)	6 (75.0)	2 (25.0)	0.66
Others	7 (5.9)	5 (71.4)	2 (28.5)	0.85
**Symptoms** (*n* (%))				
None	77 (65.8)	34 (44.2)	43 (55.8)	0.35
Otorhinolaryngologic symptoms^a^	15 (12.8)	7 (46.7)	8 (53.3)	0.85
Respiratory symptoms ^b^	7 (6.0)	4 (57.1)	3 (42.9)	0.51
Digestive disorders^c^	3 (2.6)	1 (33.3)	2 (66.7)	0.71
General signs^d^	15 (12.8)	4 (26.7)	11 (73.3)	**0.047**
**Baseline Ct values: mean (SD)**				
**All**		29.0	30.0	0.653
**Clinical form**				
Symptomatic form	27.7 (12.9)	26.1 (14.2)	29.6 (11.5)	0.421
Asymptomatic form	30.37 (9.63)	30.7 (9.2)	30 (10.1)	0.787
**Symptoms**				
None	30.5 (9.4)	30.8 (9)	30.2 (9.8)	0.805
Otorhinolaryngological symptoms^a^	30 (9.3)	26 (13.1)	33.1 (3.5)	0.166
Respiratory symptoms^b^	33.6 (2.88)	32.5 (3.5)	34.3 (2.8)	0.565
General signs^d^	24.4 (16.3)	24 (16.8)	25.5 (17.2)	0.884
**Medical history**				
No	30.3 (9.6)	28.8 (11.2)	31.3 (8.2)	0.322
Yes	27.3 (13.3)	28.3 (12.5)	26 (14.7)	0.639

*CT* Cycle threshold, *SD* Standard deviation

^a^Otorhinolaryngological symptoms (anosmia, ageusia, flu syndrome, sore throat)

^b^Respiratory symptoms (cough, dyspnea, chest pain)

^c^Digestive disorders (diarrhea, vomiting)

^d^General signs (asthenia, stiffness, myalgia, headache, pain)

### Effect of intervention on duration of viral ribonucleic acid (RNA) conversion

**Fig. 3:**
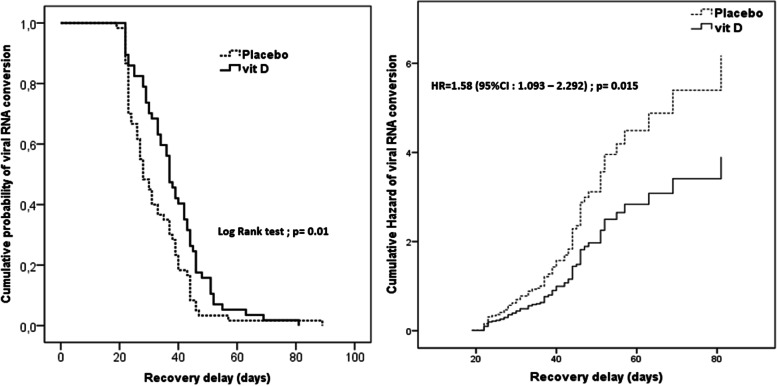
The recovery delays according to vitamin D supplementation (**A**) and hazard ratio (**B**). **A** The median duration of viral RNA conversion was 37 days (IQR: 29–45.50) in the intervention group and 28 days (IQR: 23–39) in the placebo group (*p*=0.010). B: HR was 1.58 (95% CI: 1.093–2.292, *p*=0.015). CI, confidence interval; HR, hazard ratio; RNA, ribonucleic acid; Vit D, vitamin D

The median duration of viral RNA conversion was 37 days (IQR: 29–45.50 days) in intervention group and 28 days (IQR: 23–39 days) in the placebo group (*p*=0.010). HR was 1.58 (95% CI: 1.093–2.292, *p*=0.015) as shown in Fig. 3.

**Fig. 4:**
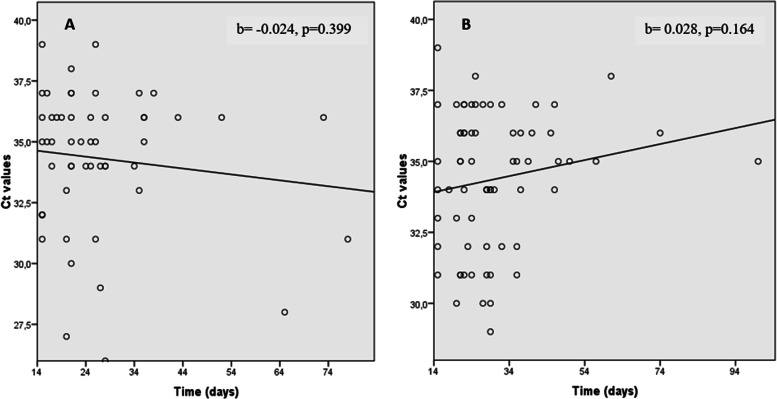
Scatter plots and linear regression of the cycle threshold (Ct) value distribution over time in the placebo group **A** and in the vitamin D supplementation group **B**. **A** Placebo group: *b*= −0.024 (SE=0.029), *r*=−0.118, *p*=0.399). **B** VDs group *b*= 0.028 (SE=0.02), *r*=0.172, *p*=0.164. Ct values revealed a stable trend over time in both intervention groups. b, slope of linear regression; SE, standard error; VDs, vitamin D supplementation

Monitoring Ct values revealed a stable trend over time in both intervention group (*b*= 0.028 (SE=0.02), *r* = 0.172, *p*=0.164) and control group (*b*= −0.024 (SE=0.029), *r* = −0.118, *p*=0.399). The dispersion of Ct values over time in the two groups is shown in Fig. 4.

### Ancillary analysis

At one year of follow-up, persistent COVID-19 symptoms were noted in 34.5% and 38.9.% of patients who received the VDs and the placebo, respectively (*p*= 0.76). A second COVID-19 infection after the first episode was noted in 10% of patients assigned to the VDs group and in 0% of patients in the placebo group (*p*=0.158).

## Discussion

The main results of the present study including 117 COVID-19 patients with mild-to-moderate symptoms and a positive control RT-PCR on the 14th day following the confirmation of the diagnosis are that the recovery delay was longer in the VDs group than in the placebo group. To the best of the author’s knowledge, the present study was the first study which has explored the impact of VDs on recovery delay among COVID-19 patients in Tunisia. Vitamin D was not associated to a shortening recovery delay when administered for patients having a positive test on the 14th day; conversely, the median duration of RNA viral conversion was significantly longer in the intervention group than in the placebo group. We have included a random sample of patients having COVID-19 confined in the national center. Participants’ characteristics at baseline were comparable in the two groups. They were randomly allocated in group intervention or placebo. A recent meta-analysis concluded that serum vitamin D levels could be implicated in the COVID-19 prognosis [19]. Another systematic review reported that the evidence is currently insufficient to support the routine use of vitamin D for the management of COVID-19 [20].

In our study, no beneficial effect of VDs on recovery delay among COVID-19 patients was noted. The median duration of viral conversion was longer in the VDs group than the placebo group. The study results were consistent with those found in the literature. In fact, no significant association between the vitamin D level and the risk of COVID-19 infection [21, 22] or the severity of this infection [11, 23] was demonstrated.

However, several studies highlighted a beneficial effect of vitamin D among COVID-19 patients [24–26]. A clinical trial performed in India revealed that a high dose of VDs decreased significantly the fibrinogen inflammatory biomarker among asymptomatic or mildly symptomatic patients with vitamin D deficiency [24]. Furthermore, a 5000 IU VDs reduce significantly the average days to resolve cough and ageusia among patients with mild to moderate COVID-19 [26]. SARS-CoV-2 positivity and worse outcomes and prognosis were inversely associated with serum vitamin D levels [19, 27]. It suggested that serum vitamin D levels could predict the severity of the COVID-19 disease and the deficiency was significantly associated with a higher rate of the intensive care unit admission and a higher mortality rates in hospitalized COVID-19 patients [28]. Our results can be explained by the fact that viral RNA conversion may be affected by other factors, not except VDs. Several studies found that the older age [26, 29], the use of masks, and COVID-19 symptoms [17] were significantly associated with the delay of RNA viral conversion.

In our study, we analyzed the Ct value which represents the number of replication cycles that are needed during real-time PCR to develop the target sample above a threshold level [30]. The higher the initial concentration of a target gene in a sample, the fewer the number of required cycles to achieve the threshold level and the lower the Ct value [31]. The present study found that Ct value trends were stable in the placebo group and slightly increasing in the intervention group. A study conducted among patients admitted to the intensive care unit demonstrated that a low vitamin D level was associated with lower Ct values and the mean Ct value was significantly lower among patients with low vitamin D levels than those with normal vitamin D levels (27 and 33.6 respectively, *p*=0.02) [32].

The present study presented some limitations. The analysis of vitamin D serum levels was not done. This can be used to identify COVID-19 patients with vitamin D deficiency at baseline. Another limitation of our study was the fact that Ct values were measured on the 14th day following the confirmation of COVID-19 diagnosis for those who were asymptomatic, and on the 7th day after the disappearance of symptoms for pauci-symptomatic ones.

## Conclusion

According to the study results, vitamin D should not be prescribed to all patients infected by SARS-COV-2. The VDs had not a beneficial effect on recovery delay among COVID-19 patients.

## Data Availability

The datasets generated during and/or analyzed during the current study are available from the corresponding author on reasonable request
